# Identification of male-specific *amh* duplication, sexually differentially expressed genes and microRNAs at early embryonic development of Nile tilapia (*Oreochromis niloticus*)

**DOI:** 10.1186/1471-2164-15-774

**Published:** 2014-09-09

**Authors:** Orly Eshel, Andrey Shirak, Lior Dor, Mark Band, Tatyana Zak, Michal Markovich-Gordon, Vered Chalifa-Caspi, Esther Feldmesser, Joel I Weller, Eyal Seroussi, Gideon Hulata, Micha Ron

**Affiliations:** Robert H. Smith Faculty of Agriculture, Food and Environment, Hebrew University of Jerusalem, Rehovot, 76100 Israel; Institute of Animal Science, ARO, The Volcani Center, Bet Dagan, 50250 Israel; W.M. Keck Center for Comparative and Functional Genomics, University of Illinois at Urbana Champaign, Urbana, IL USA; Fish & Aquaculture Research Station, Dor, Hof HaCarmel, 30820 Israel; National Institute of Biotechnology in the Negev, Ben-Gurion University of the Negev, Beer-Sheva, Israel; The Nancy and Stephen Grand National Center for Personalized Medicine, Weizmann Institute of Science, Rehovot, Israel

**Keywords:** Sex-determination, Gene expression, MicroRNA, CNV, Tilapia, Embryo, *amh*, *amhy*, *cr/20β-hsd*

## Abstract

**Background:**

The probable influence of genes and the environment on sex determination in Nile tilapia suggests that it should be regarded as a complex trait. Detection of sex determination genes in tilapia has both scientific and commercial importance. The main objective was to detect genes and microRNAs that were differentially expressed by gender in early embryonic development.

**Results:**

Artificial fertilization of *Oreochromis niloticus* XX females with either sex-reversed ΔXX males or genetically-modified YY ‘supermales’ resulted in all-female and all-male embryos, respectively. RNA of pools of all-female and all-male embryos at 2, 5 and 9 dpf were used as template for a custom Agilent eArray hybridization and next generation sequencing. Fifty-nine genes differentially expressed between genders were identified by a false discovery rate of p < 0.05. The most overexpressed genes were *amh* and *tspan8* in males, and *cr/20β-hsd*, *gpa33*, *rtn4ipl* and *zp3* in females (p < 1 × 10^−9^). Validation of gene expression using qPCR in embryos and gonads indicated copy number variation in *tspan8*, *gpa33*, *cr/20β-hsd* and *amh*. Sequencing of *amh* identified a male-specific duplication of this gene, denoted *amhy*, differing from the sequence of *amh* by a 233 bp deletion on exonVII, hence lacking the capability to encode the protein motif that binds to the transforming growth factor beta receptor (TGF-β domain). *amh* and *amhy* segregated in the mapping family in full concordance with SD-linked marker on LG23 signifying the QTL for SD. We discovered 831 microRNAs in tilapia embryos of which nine had sexually dimorphic expression patterns by a false discovery rate of p < 0.05. An up-regulated microRNA in males, pma-mir-4585, was characterized with all six predicted target genes including *cr/20β-hsd*, down-regulated in males.

**Conclusions:**

This study reports the first discovery of sexually differentially expressed genes and microRNAs at a very early stage of tilapia embryonic development, i.e. from 2 dpf. Genes with sexually differential expression patterns are enriched for copy number variation. A novel male-specific duplication of *amh*, denoted *amhy*, lacking the TGF-β domain was identified and mapped to the QTL region on LG23 for SD, thus indicating its potential role in SD.

**Electronic supplementary material:**

The online version of this article (doi:10.1186/1471-2164-15-774) contains supplementary material, which is available to authorized users.

## Background

There are more than 24,000 species of fish [1]. Research on fish sex determination (SD) has provided important insight into the plasticity of the sex-determination process in vertebrates since the biology and ecology of fish is particularly diverse and provides unique examples of sex-determination mechanisms, yet they possess many of the same processes and pathways that are used in other vertebrate systems. Sex and sex ratio have been attributed to a dominant gene (*SRY* in human), gene dosage (*Drosophila*), environmental influence (Alligator), or by the ‘threshold dichotomy’ theory, that applies to a trait with contrasting phenotypes originating from multiple genes with quantitative effects [2–5]. Tilapia SD has been well studied for its potential to produce all-male progeny with enhanced growth rate due to lack of reproductive interactions in commercial ponds. However, dimorphic differences between male and female karyotpes have not been displayed [6]. A variety of evidence suggests that sex determination in tilapia is a complex trait governed by the interactions between a genetic determination and the influence of temperature [7]. The hypothesized dual sex chromosome system for tilapia species, XX-XY system for *O. mossambicus* and *O. niloticus*, and WZ-ZZ system for *O. aureus* and *O. urolepis hornorum* was adopted by Hickling [8]. The primary support for these hypotheses was obtained from analysis of sex-ratio of progeny of: i. inter-specific crosses [9]; ii. intra-specific crosses using sex-reversed individuals [10]; and iii. chromosome set manipulations through gynogenesis [11] and androgenesis [12].

The differences in the SD mechanism among closely related tilapia species and the probable influence of sex determining genes and the environment, suggest that SD should be analyzed using a markers-based QTL approach [7, 13]. However, *O. niloticus* and *O. aureus* have different sex chromosome systems and their ability to mate and produce fertile hybrids further complicates the elucidation of the SD system. Mapping QTL for SD was based on a second-generation genetic linkage map of tilapia [14]. Studies in *O. aureus, O. mossambicus*, *O. niloticus* and F_2_ family derived from *O. aureus* × *O. niloticus* cross identified QTL for SD on LG 1, 3 and 23 [15–20]. The region on LG23 affecting SD was further fine mapped using a segregating family of Nile tilapia to 1.47 Mbp harboring 51 genes including *amh* [21]. Differential expression of *amh* between genders was reported in brain and gonads from 10 days post fertilization (dpf) embryos in Nile tilapia [22]. In zebrafish *amh* has independent functions in inhibiting both steroidogenesis and spermatogenesis [23]. The complexity of SD and the limitations of QTL mapping and the candidate gene approach [24] complicate the identification of the causative genes for SD. Thus, transcriptome-wide gene expression by gender may be used for the identification of genes that are involved in SD and sex differentiation.

microRNA (miRNA) are small noncoding RNAs, about 21 nucleotides in length. Many are conserved, and may regulate up to 30% of gene expression by base-pairing to partially complementary mRNAs [25]. Recently, Huang et al. [26] published 184 miRNAs in skeletal muscle of Nile tilapia. Yan et al. [27] identified 25 conserved miRNAs in tilapia skeletal muscle using small RNA cloning. By examining the expression of nearly 250 of the most abundant rodent miRNAs, Bale and Morgan [28] identified a robust sex-specific pattern of miRNA expression in the neonatal brain. Study on mouse characterized 55 miRNA signatures in testis and ovary [29] and illustrated their importance for the proliferation of PGCs and spermatogonia [30]. Additional studies in chicken and zebrafish identified sex-specific pattern of miRNA expression in brain, embryo and gonads [31, 32]. These findings suggest that miRNAs may play a significant role in development and more specifically in SD.

The critical period of sensitivity for elevated temperature [33] or hormonal treatment [34] to induce sex reversal of Nile tilapia was determined from fertilization to 21 days post hatching. Ijiri et al. [35] detected differentially expressed genes in XX and XY bi-potential gonads during the period of 9–10 dpf. Rougeot et al. [36] applied temperature treatment on presumable all-female population embryos until hatching (2–3 dpf) and showed ~20% phenotypic sex reversal of females to males. In addition, the findings of sex-specific mortality closely after hatching indicates that the initiation and regulation of SD pathways begin during the first few days of embryonic development, i.e. <3 dpf [15]. Preliminary analysis of candidate genes for SD at 2 to 9 dpf confirmed their functionality at early embryonic development [21]. Thus, the objective of this study was to conduct a transcriptome-wide search in Nile tilapia at early embryonic development for genes and miRNA of the SD mechanism and sex differentiation.

## Methods

### Animals and tissue collection

Breeding of *Oreochromis niloticus* (Swansea stock) families used in this study was performed at the aquaculture research station Dor, Israel. To obtain all-female (XX) and all-male (XY) progeny, eggs collected from six *O. niloticus* females were artificially fertilized with milt stripped from either two hormonally sex-reversed males (ΔXX, Nile tilapia, Manzala strain) or three genetically-modified ‘supermales’ (YY, Nile tilapia, Swansea strain, Fishgen Ltd) thus creating all-female and all-male progeny, respectively [37]. For each full-sib group, a pool of 15–30 embryos were collected at 2, 5 and 9 dpf, immediately placed in RNAlater reagent (Qiagen, USA) and then stored at −20°C until RNA extraction. The remaining fish in the group were grown until the age of three months and the sex of at least 60 individuals was determined by microscopic analysis (X100 magnification) of gonadal squash. Groups with less than 95% of individuals having the same sex were not included in the experiment. At 75 dpf, five males and females from each full-sib group were sacrificed for collection of brain, gonads and liver. The experimental protocol was approved by the Animal Care Committee of ARO.

### DNA, RNA extraction and cDNA synthesis

DNA was extracted from fin samples using the MasterPure™ DNA Purification Kit (Epicentre® Biotechnologies, WI, USA) following the manufacturer’s recommended protocol. Total RNA was extracted from a pool of 5–15 deyolked embryos (mirVana™ miRNA Isolation Kit, Ambion). Synthesis of cDNA was done with SuperScript II (Invitrogen, USA) according to the manufacturer’s instructions. The quantity and quality of the RNA samples were verified using a NanoDrop ND-1000 Spectrophotometer (NanoDrop Technologies, Wilmington, DE) and an Agilent 2100 Bioanalyzer for RNA (Agilent Technologies, Palo Alto, CA).

### Agilent microarray design

**Figure 1:**
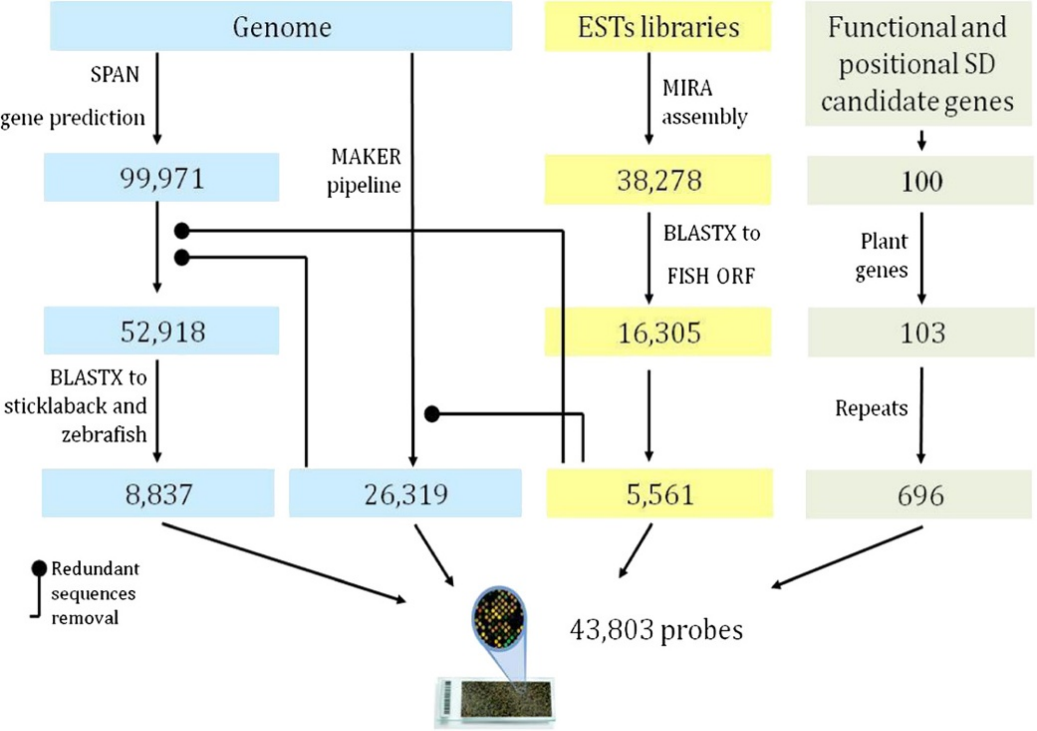
**Tilapia gene expression eArray design.** The number of probes originating from each one of three types of bioinformatic analyses is presented in different colors. Redundant sequences from different analyses were removed. Genome: Nile tilapia genome first draft sequences, SD: sex determination, FISH ORF: open reading frames of publicly available fish proteins (zebrafish, tetraodon, fugu, and stickleback).

In the absence of an expression array in tilapia we searched three bioinformatic resources of tilapia, i.e. *O. niloticus*; Broad Institute assembly (Orenil1.1; accession no. PRJNA59571), EST libraries [38] and candidate genes for SD to establish a 35,156, 5,561 and 696 different probes, respectively, that represent the transcriptome (Figure 1). Custom gene expression array was designed using eArray (Agilent Technologies, Santa Clara, CA) and 60-mer probe synthesis on a 4 × 44 k format. Our target sequences for generation of probes were based on the tilapia genome and the EST libraries. Genes were annotated by the Maker pipeline that is based on *ab-initio* gene predictions and EST evidence, and *ab-initio* SPAN gene predictions based on a full genome training set. Additional genes were identified based on assembly of tilapia EST libraries (using MIRA [39]) followed by BLASTX to fish ORF [40]. Additionally, a set of 103 positive and negative probes were designed from known genes, and were represented 6 to 7 times each on the array. Negative controls were probes representing three plant genes, whereas positive controls were probes of genes that were known to participate in sex determination and/or differentiation pathways in various species (*cyp19*, *sox9*, *amh*, *elavl1*, *dmrt1*, *foxl2*, *lhx9*, *sox14*, *msp* and *gnrh2*) and genes spanning from 1,050 to 2,488 Kbp on scaffold 102 of tilapia that were previously suggested as positional candidate genes for SD [21, 41]. The resulting eArray of 43,803 probes was used for hybridization with cDNA of 56 biological samples of predetermined gender at 2, 5 and 9 dpf of embryonic development.

### Microarray hybridization and data analysis

Two hundred ng of total RNA was labeled using the Agilent 2-color low input quickamp labeling kit according to the manufacturer’s protocols (Agilent Technology, Santa Clara, CA). Labeled samples were hybridized to a custom designed tilapia 4 × 44 K eArray containing 43,803 probes and scanned on an Axon 4000B microarray scanner (Molecular Devices, Sunnyvale, CA) at 5 μm resolution. A total of 56 biological samples at three embryonic developmental stages of 2, 5 and 9 dpf were used as template for cDNA synthesis and array hybridization. Spot finding and background correction of signal intensities were carried out using GenePix 6.1 software (Molecular Devices, Sunnyvale, CA). The microarray expression data was normalized with the Bioconductor 2.8 LIMMA package [42] using loess and aquantile normalization with a single channel analysis design. The replicated probes were represented by their median expression for analysis. The microarray data were deposited in NCBI’s Gene Expression Omnibus [43] under accession No. GSE50974.

### Statistical analysis

The normalized data of each of 43,210 different probes recorded on 56 samples were log transformed and PCA was performed by the Partek software [44] with normalized eigenvector scaling and correlation dispersion matrix. In addition each probe was analyzed by the General Linear Model (PROC GLM) procedure of SAS. The effects included in the model were slide, array, dye, gender (male or female), sire nested within gender, dpf and dam. Least square means were computed for the effects of gender. Gender was given a value of 0 for males and 1 for females. Probabilities of the differences between the “male” and “female” effects were computed based on the least squares means standard errors for each effect. The probability values were then sorted from lowest to highest, and the false discovery rate (FDR) was computed for each probe, based on the ratio of expected to observed numbers of probes for each probability value. Bonferroni probabilities taking into account multiple testing were also computed as 1- the Poisson probability to obtain zero “significant” probes for each expectation of the number of probes for each nominal probability value. Pearson correlations were computed among the 59 significant probes for gender effects at each time point for the three pair-wise combinations of dpf (2, 5 and 9).

### Functional annotation clustering

The tilapia genome is not well annotated compared to the human genome. Thus, we used the human orthologs for gene ontology analyses. The corresponding human Gene IDs were identified using NCBI BLAST. Thirty-nine out of 59 genes had identified human orthologs (Additional file 1: Table S1). The DAVID classification system [45] was performed to assess the probability of over representation of genes within the list of human orthologs of certain pathways, biological processes and molecular functions using medium to highest classification stringencies and the default of Bonferroni correction for multiple testing.

### Validation of microarray results using quantitative real-time PCR

Validation of probes was done by qPCR using RNA of three to four pools of monosex embryos, each comprising samples of 28 females or 30 males at eight daily time points from 2 to 9 dpf. Primer design was based on sequence of ~100 bp flanking each of eight probes that were submitted to “Primer3plus”program [46]. For a given probe, one of the primers in each pair was identical to the original sequence from the microarray experiment targeting the probe’s location (Table 1). The fragment’s sequence was used to BLAST search the tilapia genome to confirm its position in the genome (Additional file 1: Table S1).

**Table 1:** **Primers for gene expression validation with qPCR and** ***amh*** **PCR and sequence analysis**

Gene symbol/probe	Description	Accession number	Forward and reverse primers (from 5’ to 3’)
*tspan8*	tetraspanin-8	XM_003448079	TGTATGTTGTGGAATAGGCATCA
			GGTGATTTGTAAAGCTGTTTCG
*fcgrt*	major histocompatibility complex class I-related gene protein-like	ENSONIG00000008406	TGGTGTGAGCAAGGACTTCAT
			ACATGAACCAAAGGACTGTAAACT
*cr/20β-hsd*	carbonyl reductase-like 20 beta-hydroxysteroid dehydrogenase	XM_005473633	CCAAAATTGTTCGTTTTATTCTCG
			TTTCATTTTGATGCGTTCCA
*gpa33*	cell surface A33 antigen	XM_005466820	AATGTCCAAAAGCCAACCTAAA
			TACTACATCTGCACCTCGGAGA
*rtn4Ip11*	reticulon-4-interacting protein 1 homolog, mitochondrial	XM_003459954	GCATGTCAAGGCATCAAATAAA
			CCTCTGCTGGTTGTAAATGTGA
*26098*	hypothetical protein		TTCCTGAAGACAGTACAGTACAAAA
			CGAATTCTTCTGGTCAAGTTCTTC
zp3	zona pellucida sperm-binding protein 3 receptor-like	XM_005448435	TGTCTGTAACTACTCATTTGGATCA
			CCATTTTACAGATCCAACTTTCC
*gapdh*	glyceraldehyde-3-phosphate dehydrogenase	XM_003452690	GGCATCGTGGAAGGTCTCAT
			CATTTTACCAGAGGGCCCGT
*rpp30*	ribonuclease P protein subunit p30	XM_005471600	CCCGACTCCTATCAACGAAC
			AAAGTGACTCGCGTCTGACA
*amh*	anti-Müllerian hormone^1^	DQ257619.1	TTCTTATCGCTCCGACTTCTTC
			TAGGGCTGGTTGATATGGAATC
	anti-Müllerian hormone - exon VII^2^	XM_003451305	AGCAGCTCTAGCGGCATCCACA
			TGTGTTTTCTTTCTGCGTCCGCCA
	1^3^: 5’ UTR, exon I, intron 1, exon II	ENSONIG00000004781	AGAGGAGTCATCAGTCCAAAGC
			AGATGTCCTCCACGAAGCAT
	*2*: exon II, intron II, exon III	ENSONIG00000004781	AAGACCCCATCATCACCATC
			TTGTCTGAGCCGTAATCTGC
	*3*: exon IV, intron III, exon V, intron IV, exon VI	ENSONIG00000004781	GGAAAATCATCAGAGGGGAGT
			CTGCCGACTTCAGAACTTTT
	4: intron VI, exon VII, 3’ UTR	ENSONIG00000004781	CGGTCCCAGTGACCTATGAG
			AAGTACACGTGGTGTATTGTAATTGA
	*5:* 3’ UTR	ENSONIG00000004781	CCCCAGCATTTATAACTTTCACA
			CCTGCCTCAAGTATGCCTTT
	LM^4^: intron VI, exon VII	ENSONIG00000004781	TGTGTTTTCTTTCTGCGTCCGCCA
			AGCAGCTCTAGCGGCATCCACA

^1^qPCR validation of microarray.

^2^PCR of cDNA.

^3^set of 5 primer pairs for sequencing.

^4^Linkage mapping.

The qPCR analysis was performed in triplicates using the Fast SYBR^®^ Green Master Mix kit (Thermo Fisher Scientific, UK) according to the instructions of the manufacturer in a 17-μl reaction volume, which included 2 μl of DNA (30 ng/μl), 1 μl of each primer (10 pmol/μl), 4.5 μl of ultra-pure water, and 8.5 μl of Absolute Blue SYBER Green ROX Mix. The qPCR reaction was performed in the following conditions: 20 seconds at 95°C for enzyme activation followed by 40 cycles of 3 sec at 95°C, 30 sec at 60°C using StepOnePlus™ Real-Time PCR System. Amplification was followed by melting-curve analysis to confirm specificity of products. A standard curve was generated for each gene using serial dilutions of the specific PCR product, for the absolute quantification method. The threshold cycle number (Ct) for each tested probe was used to quantify its relative abundance. The StepOne Software v2.2.1 (ABI) was used for the calculation of the relative quantities using Glyceraldehyde-3-phosphate dehydrogenase (*gapdh*) for normalization. The relative amount of the target RNA, designated as the input amount (IA) was determined by comparison with the corresponding standard curve for each sample (User Bulletin #2 ABI PRISM7700 Sequence Detection System, Applied Biosystems). The IA values were calculated as follows: IA = [10^((Ct - intercept)/slope)^], where Ct is the cycle threshold for unknown sample. The female to male ratio of expression was computed based on the mean IA of eight daily samples of male and female embryos from 2 to 9 dpf. Pearson correlations between the female to male ratio of expression from the microarray experiment and that from qPCR was performed for eight genes (*fcgrt*, *rtn4ip1*, *CUST_26098*, *zp3*, *gpa33*, *tspan8*, *cr/20β-hsd* and *amh*) using Excel. *cr/20β-hsd* and *amh* were further characterized for their expression by qPCR in brain, liver and gonads of 75 dpf female and male fish. These genes have shown expression in brain and gonads in previous studies, and liver is used as a negative control.

### Copy number variation using quantitative real-time PCR

Determination of the relative copy number of eight probes was conducted using qPCR analysis based on genomic DNA (gDNA) template. Preliminary work was conducted on randomly selected 13 and 14 male and female gDNA samples, respectively. Gene copy number was normalized to the reference gene, Ribonuclease P protein subunit p30 (*rpp30*) that was used as a reference gene in human CNV studies [47]. Additionally, five offspring of crosses between two dams and five sires were analyzed for relative copy number.

### *amh*sequencing and linkage mapping

Full length *amh* gene and the 2,000 bp flanking it were amplified in ~1,000 bp fragments with primer design based on the Ensembl sequence scaffold GL831234.1:1,686,017-1,697,999 (Table 1). PCR templates included DNA of XX female, XY and YY males. PCR reaction protocol was according to the manufacturer’s instructions using the high-fidelity BIO-X-ACT Long DNA polymerase (Bioline, London, UK). PCR products were separated on agarose gels and stained with ethidium bromide. The DNA fragments were visualized with UV light and excised from the gel. DNA fragments were purified with the DNA gel extraction kit (Millipore, Bedford, MA) and then sequenced on 3730 DNA analyzer (Applied Biosystem, USA). Sequence trace files were assembled and analyzed with the GAP4 package [48]. Linkage mapping for SD was performed by genotyping the *O. niloticus* mapping family for *amh* and *amhy* (Table 1) and microsatellite *UNH898* [21]. PCR fragments were subcloned separately into pPCR TOPO vector (Invitrogen, USA) and sequenced in sense and antisense directions.

### Small RNA sequencing

Six small RNA libraries were prepared for ‘super’ pools of full-sib embryos of males and females at 2, 5 and 9 dpf, that were used for the microarray experiment, with Illumina’s ‘TruSeq’ Small RNA Sample Prep kit. The libraries were size-selected to 18–33 nucleotides fragments, quantitated by qPCR and divided into two samples that were sequenced on separate lanes for 41 cycles on a HiSeq2000 using a TruSeq SBS sequencing kit version 3. The sequences were analyzed with Casava1.8 (pipeline 1.9) yielding between 21 to 32 million sequences per sample.

### Identification of miRNAs

The sequence reads were mapped to the tilapia genome and analyzed by miRDeep 2.0.0.5- mapper script, an algorithm based on the miRNA biogenesis model [49]. It aligns sequencing reads to potential hairpin structures in a manner consistent with Dicer processing, and assigns log-odds scores to estimate the probability that hairpins are true miRNA precursors. The output of this analysis is a scored list of miRNAs that passed the stringent score cut-off of four, which reflects a signal-to-noise ratio greater than 12. Expression levels were normalized to the size of each of the six libraries (reads per millions). The miRNAs were searched against and submitted to miRbase for miRNA gene name assignment. Novel miRNAs in tilapia were assigned “oni-mir number”. miRNA data was deposited in miRbase.

### Differential expression of miRNAs between genders

We used the miRDeep 2.0.0.5 quantifier script, a module that maps the deep sequencing reads falling into an interval of two nucleotides upstream and five nucleotides downstream of the mature/star sequences of the predefined miRNA precursors, to estimate the expression of the corresponding miRNAs in each sample. Read counts of mature and star sequences of ≥ four read counts were log transformed and the deviations between genders were analyzed in each of 2, 5 and 9 dpf, separately. The FDR was calculated assuming a normal distribution, i.e. comparing the realized number of deviations ≥ 4 standard deviations to those expected by random at p = 3.17 × 10^−5^. Thus, deviations between genders ≥ 4 standard deviations corresponding to a FDR of 1.5% were considered statistically significant.

### Detection of gene hosts for differentially expressed miRNAs

Gene hosts were searched for the conserved up-regulated mir-21 and mir-218 in males, using BLASTN with their precursor sequences against the sequence data of vertebrate species (Ensembl). ESTs from vertebrates were assembled in the vicinity of the miRNAs to identify their gene hosts. Tissue specific pattern of mRNA expression in zebrafish was analyzed for the identified gene hosts using BioGPS [50].

### miRNA target prediction analysis

Nine miRNAs differentially expressed by gender were explored for potential gene targets represented by their mature sequences (3p/5p) in 59 differentially expressed genes between genders. The 3’UTR sequences of the 59 genes were downloaded from Biomart in Ensembl database. When the 3’UTR was not available, 2,482 bp downstream was used. This length is the third quantile of the known tilapia 3’UTR. Prediction of potential gene targets for miRNA was performed with RNAhybrid and miRanda software [51, 52]. Forty four gene targets that were predicted by both algorithms were considered potential miRNA gene targets.

## Results

Identification of genes and miRNA affecting SD were based on comparative analysis between genders using the same biological samples, i.e. RNA of tilapia embryos of predetermined gender, from 2 to 9 dpf.

### Analysis of differentially expressed genes between genders and CNV

Principal component analysis (PCA) for the microarray data showed that three factors explained 40, 20 and 9% of the total variance. Individual samples of gender by dpf, relative to the three factors are plotted in Additional file 2: Figure S1. There was a clear distinction between males and females except for two female samples at 2 and 9 dpf which were close to the male cluster. The effect of gender was highly correlated with the first factor, while the effect of dpf was correlated with the second factor. Correlations with the third factor were low for all effects considered.

**Figure 2:**
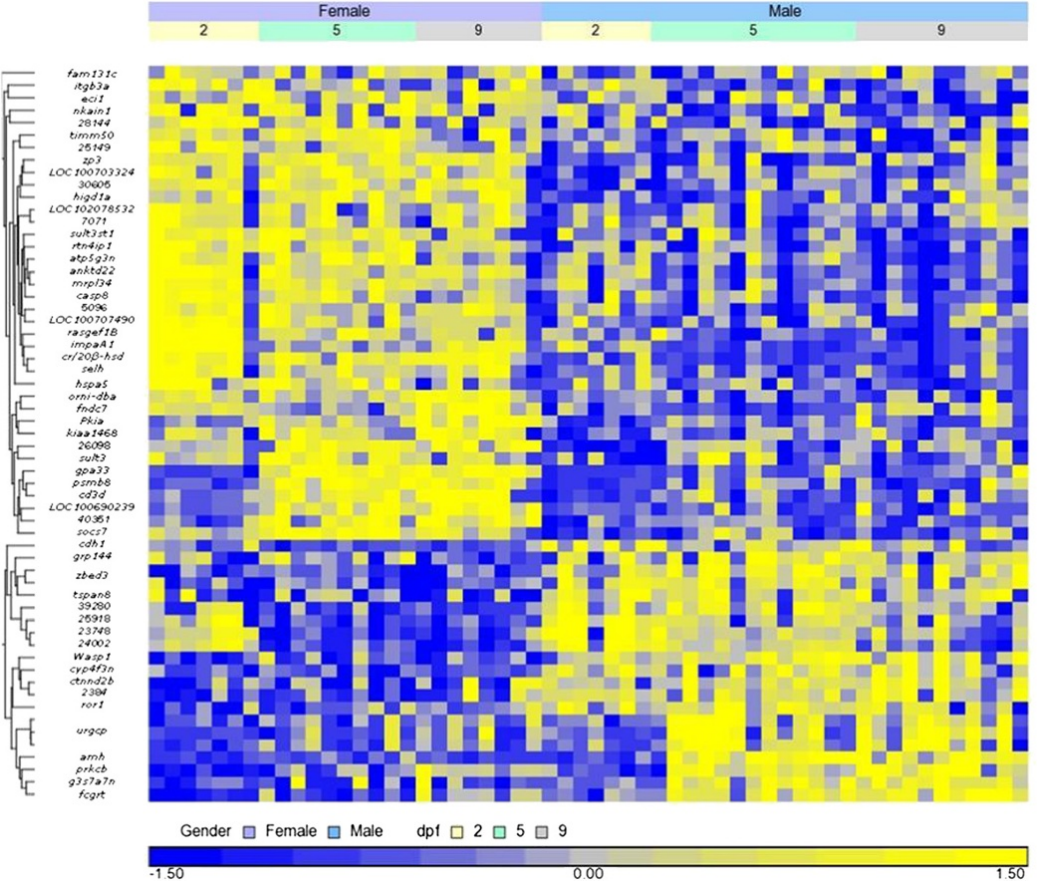
**Clustering of differentially expressed genes by gender and dpf.** Fifty nine differentially expressed genes between males and females at 2, 5 and 9 dpf, at false discovery rate of p < 0.05, were clustered. The Z-score represents the number of standard deviation units of genes’ expression value from the mean (defined as 0). Blue and yellow colors indicate up and down expression, respectively. Vertical bars represent the clustering of genes based on expression profiles. *urgcp* and *zbed3* genes appear more than once and represent different probes at multiple locations in the genome. Probe numbers begin with the prefix “CUST_”. Annotations of probes are presented in Additional file 1: Table S1. LOC numbers are NCBI IDs.

The FDR as a function of nominal significance values and number of significant probes is plotted in Additional file 3: Figure S2. There were 59 genes differentially expressed between genders significant with an FDR of 0.05, which corresponded to nominal probability of 10^−5^. Fold change values between genders ranged from 1.2 to 4.2. The gene names, their annotations, least squares means by gender as fold change between genders (FC) and potential relevance to SD and apoptosis/immune response are presented in Additional file 1: Table S1. Hierarchical clustering of all differentially expressed genes shows that only one third of them were highly expressed in males (Figure 2). The expression profiles of these genes were similar across the three time points of 2, 5 and 9 dpf, with correlations exceeding 0.79. Nevertheless, there is a tendency of earlier differentially expressed genes in females (2 dpf) than in males (5 dpf). For 39 of the genes, orthologs in human were found with full annotations. Functional annotation clustering indicated a significant enriched cluster of the immune response containing four genes e.g., *psmb8*, *fcgrt*, *gas7a7* and *zp3* (DAVID enrichment score 1.56; p < 0.01). Ten genes were involved in pathways known to affect SD, and more than half of the genes (22) were involved in apoptosis (Additional file 1: Table S1).

**Figure 3:**
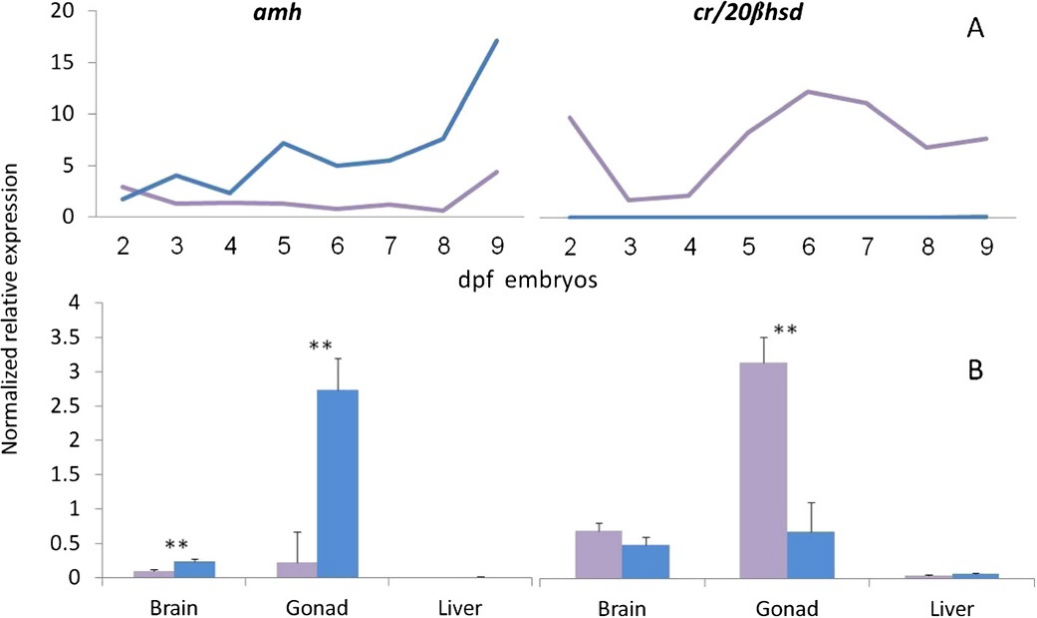
***amh*** **and** ***cr/20β-hsd*** **normalized relative expression.** Gene expression by qPCR is presented for males (blue) and females (purple) in 2 to 9 dpf embryos **(A)**, and in brain, testis/ovary and liver at 75 dpf **(B)**. Deviation bars represent standard errors and asterisks represent the level of significance for sex-specific expression differences: **p ≤ 0.001.

The genes with the most significant sexually dimorphic expression patterns were: carbonyl reductase-like 20β-hydroxysteroid dehydrogenase (*cr/20β-hsd*; Fold change (FC) 3.5; p = 1 × 10^−17^), reticulon-4-interacting protein 1 homolog *(rtn4ip1;* FC 2.5; p = 1 × 10^−17^*),* tetraspanin-8 (*tspan8*; FC 0.2; p = 1 × 10^−16^), inositol monophosphatase 1 (*impa1*; FC 2.8; p = 1 × 10^−14^), zinc-finger bed domain containing 3 (*zbed3*; FC 0.6; p = 1 × 10^−12^) and anti-müllerian hormone (*amh*; FC 0.5; p = 1 × 10^−9^) (Additional file 1: Table S1). *cr/20β-hsd*, *rtn4ip1* and *impa1* were highly expressed in females, and *tspan8, zbed3* and *amh* were overexpressed in males. The daily expression by qPCR of *amh* and *cr/20β-hsd* in 2 to 9 dpf embryos is presented in Figure 3A demonstrating the overexpression in males and females, respectively. The expression of *amh* and *cr/20β-hsd* by qPCR in brain, liver and gonads of tilapia at 75 dpf is presented in Figure 3B. *cr/20β-hsd* and *amh* genes’ overexpression were validated in the respective gonads, i.e. ovary and testis, while *amh* was also highly expressed in male brain.

**Figure 4:**
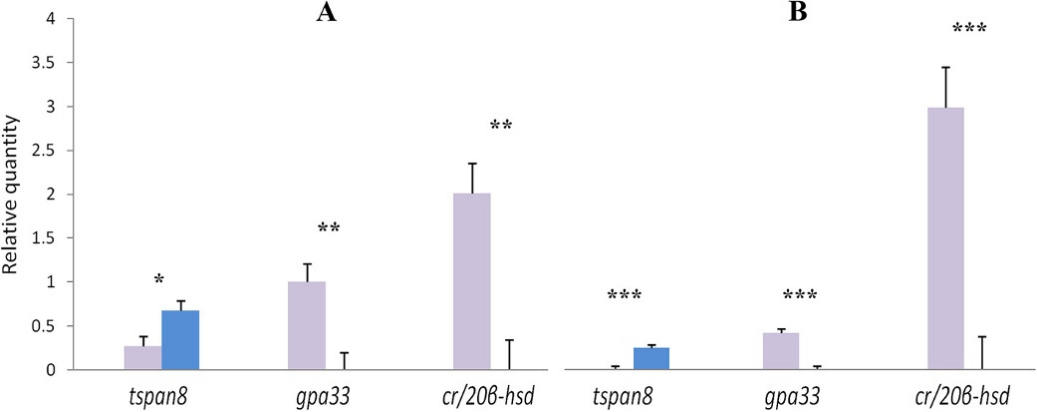
***tspan8, gpa33*** **and** ***cr/20β-hsd*** **copy number variation.** Normalized relative genomic quantity by qPCR is presented for males (blue) and females (purple) for analysis of copy number variation. **A** and **B** represent crosses involving different dams. Deviation bars represent standard errors. Asterisks represent the level of significance for sex-specific expression differences: *p ≤ 0.05; **p ≤ 0.01 and ***p < 0.001.

There were 23 probes with experimental-wise Bonferroni probabilities < 0.05. Eight out of the 23 genes were sampled for validation by qPCR. Expression data for microarray and qPCR are given in Table 2. A correlation of 0.8 for mean expression ratio between genders was obtained between the microarray and qPCR, indicating a high rate of validation. Nevertheless, melting curve analysis showed multiple amplified fragments that were specific to one of the genders for a few of the genes, indicating potential copy number variation. Further analysis by qPCR based on gDNA of males and females for four of the eight genes, *amh*, *cr/20β-hsd, tspan8* and *gpa33,* demonstrated significant copy number differences between genders. Figure 4 shows that *tspan8* had more genomic copies in males while *cr/20β-hsd* and *gpa33* had more genomic copies in females. All four genes showed direct correlation between number of copies and expression level.

**Table 2:** **Gene expression validation of microarray results**

				qPCR				
Over expressed in	Gene symbol	Microarray		cDNA		Multiple Tm peaks^3^	gDNA	
		FC^1^	p-value	FC	p-value		FC	p-value
Male	*amh*	0.5	6 × 10^−9^	0.27	0.002		0.75	7.4 × 10^−4^
	*tspan8*	0.2	4.4 × 10^−16^	0.0001	5 × 10^−11^		0.0013	1.2 × 10^−4^
	*fcgrt*	0.5	6.5 × 10^−8^	ND^**2**^		√	ND	
Female	*cr/20β-hsd*	3.5	1.0 × 10^−17^	352	2.8 × 10^−11^	√	669	0.002
	*gpa33*	3.4	6.0 × 10^−9^	88.7	2.6 × 10^−5^	√	15.8	3.3 × 10^−6^
	*rtn4Ip1*	2.5	1.0 × 10^−17^	2.1	0.0001		ND	
	*CUST_26098*	4.2	1.6 × 10^−7^	4.46	0.004		ND	
	*zp3*	2.6	3.0 × 10^−9^	2	0.004		ND	

Mean ratio of female to male gene expression by qPCR from cDNA of embryos from 2 to 9 dpf is presented. The qPCR analysis of genomic DNA (gDNA) was applied for indication of copy number variation.

^1^FC: fold change of female to male expression: LS means for Microarray and means for qPCR based on cDNA of embryos from 2 to 9 dpf.

^2^ND: not determined.

^3^Multiple TM peaks resulting from melting curve analysis.

### Identification of male specific *amh*duplication

**Figure 5:**
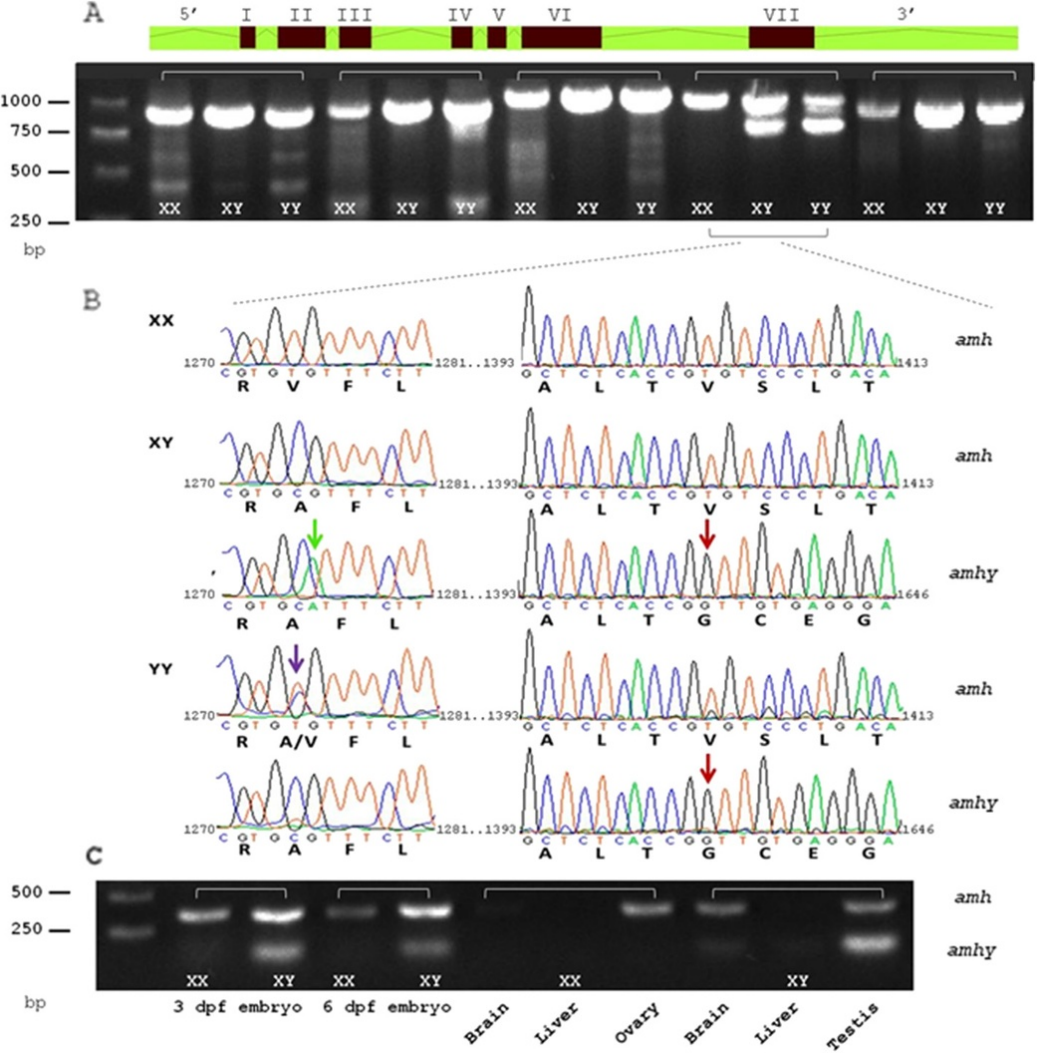
**Identification of Y-linked** ***amh*** **duplication. (A)** Schematic illustration of the full length *amh* gene. Lines shaded with green, introns; Red boxes, exons; the Roman numerals outside the boxes indicate exon number. Four sets of PCR genomic fragments are presented under the respective parts of the gene for XX, XY and YY DNA samples. **(B)** DNA sequence traces of *amh* and *amhy* exon VII of three unrelated individuals: XX female, XY and YY males (GenBank: HG518783-7). Capital letters under the traces denote the deduced capable of encoding amino acids. Purple arrow, SNP in nucleotide position 1,274 of the full length *amh* gene in YY individual; green arrow, A > G substitution in nucleotide 1,275 of *amhy* between XY and YY individuals; red arrows, deletion starts in *amhy* from nucleotide position 1,403*.* **(C)** PCR for exon VII from cDNA of male and female 3 and 6 dpf embryos, brain, liver, and gonads.

As a candidate gene for SD, *amh* was represented by five different probes on the eArray. Analysis of the microarray data showed differential expression between genders only for one of the probes that was located upstream to exon VII. This probe was highly expressed in males. Thus, we amplified and sequenced the full length *amh* gene using four PCR amplicons of about 1,000 bp each (Figure 5A, primers on Table 1). Length of amplified products was similar for both genders for the first three PCR amplicons covering exon I to VI. In the fourth amplicon we identified in both genders a fragment of 1,048 bp containing exon VII and possibly an additional somewhat smaller fragment attached to it. However, an additional fragment of 815 bp was found only in males and was termed *amhy*.

**Figure 6:**
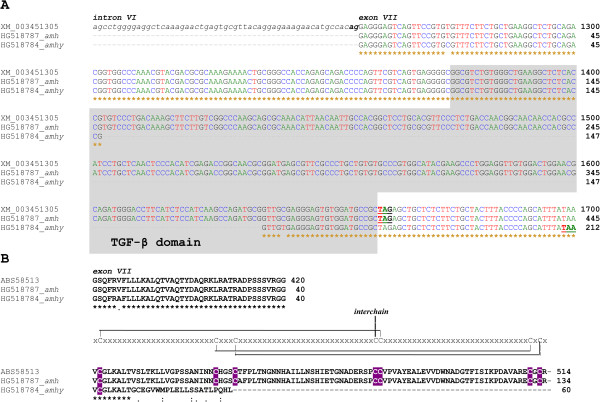
**Genomic sequences and predicted polypeptides of** ***amh*** **and** ***amhy*** **genes.** The coding region of the 7th exon of the *AMH* gene is aligned with sequences of XX-female *amh* and YY-male *amhy*. Sequences derived from GenBank entries were aligned (Accession Nos. XM_003451305, HG518787, HG518784 for *amh* reference gene, female *amh* and male *amhy*, respectively). Above the sequences, Roman numerals label the genomic element of the gene. Asterisks below the sequences denote identical residues in all three sequence submissions. Dashes mark gaps introduced by the alignment program. The shadowed regions localize the TGF-β domain. Numbers indicating the position of the last residue within the GenBank entry are added at right ends of rows. **(A)** Genomic sequences: The end of intron 6 is shown in lower-case italic letters and the last two bases of the acceptor splice site (ag) are in bold type. The in frame stop codons (TAG or TAA) are in bold and underlined type. **(B)** Predicted polypeptides: Below the sequences, conserved substitutions are indicated by two dots; and semi-conserved substitutions are indicated by dots. Cysteine residues that form disulfide bonds according to TGF-β family signature (PROSITE PDOC00223) are in white against purple background and this signature layout is delineated above the TGF-β domain sequence.

Sequencing of the two types of fragments revealed nearly identical sequences to *amh* exon VII with a deletion of 233 bp in *amhy* (Figure 5B). We sequenced the *amh* and *amhy* exon VII for XX, XY and YY unrelated individuals. The purple arrow indicates nucleotides T and C at position 1,274 of the full length *amh* in XX and XY, respectively, with both alleles present in YY. The green arrow indicates an A > G substitution in nucleotide 1,275 of *amhy* between XY and YY individuals. In *amhy*, nucleotide G was found at position 1,403 as opposed to T in *amh* in both XY and YY individuals as pointed by red arrows, with lack of alignment thereafter, indicating the start of the deletion which corresponds to transforming growth factor beta (TGF-β) binding domain (Figure 6A). Capability of translation of exon VII in *amhy*, as compared to *amh*, indicates a potential deletion of 86 amino acids and addition of 21 amino acids, due to a reading frame shift and disruption of a stop codon (Figure 6B). The potentially translated protein based on the partial *amhy* sequence exhibits at its end 12 of the 21 predicted additional amino acids.

PCR amplification with internal primers designed based on *amh* exon VII, on cDNA of male and female 3 and 6 dpf embryos, brain, liver and gonads, showed the *amh* fragment of 442 bp in all samples except liver, whereas the *amhy* smaller fragment of 209 bp was detected in male embryos, brain and testis but not in female embryos, brain and ovary (Figure 5C). Interestingly, the intermediate fragment that may be observed in Figure 5A (from DNA) do not appear in Figure 5C (from RNA). Cloning the three fragments and subsequent sequencing resulted in full length highly readable sequences. Different clones of the intermediate band resulted in sequences that were identical to those of either the higher or lower band, thus indicating that there is no a novel sequence underlying the intermediate band, but an artifact combining both the higher and lower fragments that share similar sequences.

Linkage mapping analysis showed full concordance between UNH898 on LG23, which is the closest microsatellite marker to the QTL for SD [16, 21], *amh* [41] and *amhy*. Both male-associated-allele of UNH898 and *amhy* fragment were present in all 61 males and absent in all 29 females, thus indicating that *amhy* is localized to LG23 at the SD region.

### Analysis of differentially expressed miRNAs between genders

The 171.2 million reads from the small RNA sequencing experiment were uploaded to miRDeep2 software which processes reads and using the Mapper script maps them to the reference genome for miRNA detection based on their biogenesis model. We discovered 578 miRNA precursors that passed the stringent score cut-off of four, which reflects a signal-to-noise ratio greater than 12 in tilapia and submitted them to miRBase.

We then ran the quantifier script to determine and normalize the number of reads of predefined miRNA precursors, indicating the expression of the corresponding miRNAs in each sample. Read counts by gender of 704, 668 and 636 mature and star sequences of ≥ 4 reads in 2, 5 and 9 dpf, respectively, were log transformed and analyzed for expression abundance between genders. The distributions of deviations between genders were analyzed separately in each of 2, 5 and 9 dpf and were approximately normal as exemplified for 9 dpf in Additional file 4: Figure S3. Nine sexually differentially expressed miRNAs with deviations between genders ≥ 4 standard deviations were obtained. This stringent criterion corresponds to an FDR of 1.7% by comparison to the number of miRNAs that are expected purely by chance with >4 standard deviations in a normal distribution. The nine differentially expressed miRNAs by gender are presented in Table 3. The miRNAs ranged in expression abundance between genders by approximately two to 10-folds, and were consistent across time points. Three miRNAs were up-regulated in females and six miRNAs in males. Only four of the nine miRNAs had conserved annotated names, i.e. mir-21, pma-mir-4585, bmo-mir-2779 and mir-218. Two miRNAs were independently found in two of the three time points while two others were found in all three. Thus, given the low FDR level and the independent detection of half of the miRNAs at multiple time points with stable differential expression by gender, the nine differentially expressed miRNAs may be considered reliable. Interestingly, most of the up-regulated miRNAs in males were at 2 and 5 dpf and those up-regulated in females were at 5 and 9 dpf.

**Table 3:** **miRNA differential expression between genders**

#miRNA^1^	Overexpressed in dpf	2	5	9
miR-218	Male	0.4		
pma-miR-4585		0.2	0.5	
oni-miR-E224		0.1	0.3	0.4
oni-miR-E622		0.4	0.5	0.6
oni-miR-E218			0.5	
mir-21			0.6	
oni-miR-E255	Female	3.8		
oni-miR-E304			2	2
bmo-miR-2779				1.7

miRNA are displayed with female to male normalized expression at 2, 5 and 9 dpf with ≥ four standard deviations of fold change expression in a normal distribution.

^**1**^miR: expressed 3p/5p mature sequence from miRNA precursors.

### Analysis of gene targets for differentially expressed miRNAs between genders

The 3’ UTR of the 59 genes differentially expressed between genders in the microarray experiment (Figure 2) were tested as potential targets for nine miRNAs that were differentially expressed between genders (Table 3). A total of 44 predicted gene targets were identified and presented by gender in Table 4. For each miRNA and gene target combination, concordant and discordant relationship is displayed by plus and minus symbols, respectively. Concordance is called for an up-regulated miRNA and its down-regulated putative gene target. Only one of the miRNAs e.g., pma-mir-4585 that was up-regulated in males, showed significant perfect inverse correlation of expression pattern with its six targeted genes; *cr/20β-hsd*, *psmb8*, *rtn4ip1*, *casp8*, *atp5g3* and an unannotated gene, that were down-regulated in males. Moreover, the first gene is known to activate female determination. This miRNA was up-regulated in male vs. female embryos at both 2 and 5 dpf. At 9 dpf it was up-regulated by only three standard deviations and thus was not marked as differentially expressed at 9 dpf in Table 3.

**Table 4:** **Differential expression between genders of miRNA and their gene targets**

Gene	miRNA^1^		Overexpressed in^2^								
			Male						Female		
Probe^3^	Gene symbol^4^	Down-regulated in	miR-218	oni-miR-E218	oni-miR-E224	miR-21	pma-miR-4585	oni-miR-E622	bmo-miR-2779	oni-miR-E255	oni-miR-E304
42507	*fcgrt*	**Female**			−	−		−			
86	*amh*		*−*		−			−			+
41041	*urgcp*			−	−	−		−			
38100	*ctnnd2b*		*−*		−	−					
25051	*g3s1a18*										
25051	*g3s7a7*				−						
21001	*prkcb*										
41366	*zbed3*		*−*						+		
41366	*7813*		*−*		−						
41366	*5031*		*−*	−							
41052	*urgcp*		*−*		−	−		−		+	
21060	*cyp4f3*		*−*	−	−			−			
14085	*wasp1*					−					
22935	*urgcp*				−	−		−			+
27670	*zebd3*		*−*						+		
25225	*cdh1*										
39928	*gpr144*				−	−		−		+	+
16862	*ror1*		*−*					−			
28545	*fam131c*	**Male**	*+*							−	
10109	*eci1*			+		+					
5193	*itgb3a*					+					
13137	*timm50*		*+*	+	+	+					
555	*fndc7*					+		+			
35393	*nkain1*							+			
6825	*kiaa1468*				+						
7935	*rasgef1b*		*+*			+		+			
452	*higd1a*				+	+		+			
5723	*sult3*							+			
30073	*casp8*						+				−
23873	*orni-dba*							+			
20878	*hspa5*				+						
31928	*socs7*							+			
9238	*atp5g3*				+		+				
3491	*LOC100703324*		*+*		+						
35899	*LOC100707490*		*+*				+				
800	*mrpl34*					+		+			
23235	*psmb8*						+				
13679	*ankrd22*					+		+		−	
24221	*LOC100690239*							+			
5738	*sult3st1*							+			
25471	*rtn4ip1*						+				
1524	*zp3*							+			
9317	*gpa33*				+			+			
1135	*cr/20β-hsd*		*+*				+				
Rate of concordant target sites			6/15	2/5	7/17	8/15	6/6^*^	13/21	2/2	2/4	3/4

Concordant and discordant differential expression is displayed for nine miRNAs with their forty four differentially expressed between genders predicted gene targets by a false discovery rate of p < 0.05.

^1^miR: expressed 3p/5p mature sequence from miRNA precursors.

^2^Target recognition sites for miRNAs are represented by concordant (+) or discordant (−) differential expression, respectively, assuming that up-regulated miRNAs down-regulate their target sites [74].

^3^Probe numbers begin with the prefix “CUST_”.

^4^Gene symbol based on NCBI, ENSEMBL or tilapia LOC no. *urgcp* and *zbed3* genes represent different probes at multiple locations in the genome.

^*^The probability that the rate of concordant target sites should occur by chance is: (1/2)^6^ = 0.015.

### Analysis of gene hosts for differentially expressed miRNAs between genders

Two of the nine differentially expressed miRNAs were up-regulated in males and highly conserved among vertebrates. Therefore their gene hosts could be determined by across species genomic analysis. mir-21 was identified in tubulin delta 1 (*tubd1*), and mir-218 was found in the 3’ region of developmentally regulated GTP binding protein 1 (*drg1*).

## Discussion

The main objective in the present study was to identify genes and miRNAs that were differentially expressed between genders before gonad formation. Since differences in gene expression were previously detected in the bi-potential gonads at 9–10 dpf, we analyzed the embryos at 2, 5 and 9 dpf, which are equivalent to the developmental stages of brain differentiation, hatching and late larva period, respectively [53].

Fifty-nine genes were differentially expressed between genders based on an FDR of 0.05. Correlations of expression patterns between genders were 0.85 and 0.79 between 2 dpf and 5 and 9 dpf, respectively. The correlation between the latter two stages was 0.95. Thus, the somewhat lower correlation of expression between 2 dpf and later embryonic stages may indicate partial transcription at 2 dpf of maternal RNAs stored in oocytes. Most of the detected genes are known to play a role in vertebrate SD and apoptosis*.* Functional annotation clustering indicated a significant enriched cluster of the immune response containing four genes: *psmb8*, *fcgrt*, *gas7a7* and *zp3*. A recent study detected *sdY* as a sex determining gene in rainbow trout that evolved from an immune related gene [54]. In addition, we found 22 genes that were associated to apoptosis pathways. This is in accordance with previous studies, suggesting that evolutionary conserved genes in the immune system and apoptotic cell death processes may also play a role in this early stage of differentiation and SD [55, 56]. Apoptotic pathways are known to be part of sex differentiation in zebrafish [56]. Furthermore, elimination of the Müllerian duct, the primitive female reproductive tract, is triggered by *amh* and mediated also by apoptosis in mammalian sexual differentiation of male [57]. Thus, apoptosis pathways may be involved in SD or sex differentiation of Nile tilapia.

The *amh* gene, also called Müllerian inhibiting substance, is a member of the TGF-β family that is a key player in cell proliferation, differentiation and apoptosis [58]. It is secreted by Sertoli cells and is responsible for the regression of Müllerian duct during male fetal development in mammals, birds, and reptiles [59]. This gene is also a positional candidate gene due to its location in the central region of the SD QTL on LG23 [16, 21]. Fifty-one genes were positional candidates in the 1.47 Mbp critical region of the SD QTL on LG23 in tilapia [21], but only *amh* was differentially expressed in male embryos and testis. A thorough characterization of the gene showed two SNPs in *amh* exon VII among the three unrelated individuals tested, and a novel duplication in males. This unique male-specific copy, denoted *amhy,* has a deletion of 233 bp of the TGF-β domain, and is therefore not capable of encoding the corresponding 86 amino acids. However, a capability of encoding additional upstream 21 amino acids emerged, due to a reading frame shift and disruption of a stop codon*.* Recently, Y-linked *amh* duplication was identified with a 577 bp insertion in intron 3, and a critical role in SD of Patagonian pejerrey [60]. In addition, SNP in the kinase domain of anti-Müllerian receptor type II *(amhr2)* was found to be associated with SD in fugu [61]. Apparently all other candidate genes that were represented on the array including *cyp19 aromatase*s, *dmrt1*, *elavl1*, *gnrh*, *msp*, *sox9*, *sox14*, *lhx9* and *foxl2* were not sexually differentially expressed at the early embryonic development; although they are highly expressed in either ovary or testis of tilapia [41]. This may reflect the pivotal early control of *amh* in SD, and the role of additional downstream genes participating in formation and function of the gonads. However, the mechanism through which a duplicated copy of *amh* lacking its regulatory region may lead to male determination remains unknown. Furthermore, *amhy* could also be an ancient sex determining gene with no effect on male determination in the contemporary SD mechanism of Nile tilapia.

Additional genes that were highly expressed in males were *tspan8, zbed3, wasp1, cyp4f3* and *prkcb. tspan8* and *zbed3* were reported as overexpressed in testis, interacting with axin protein thus activating Wnt/β-catenin signaling and TGF-β/BMP pathways, respectively [62, 63]. *cyp4f3* was found to be necessary for efficient male mating in *Drosophila melanogaster* and *prkcb* modulates SMAD-dependent TGF-β signaling [64, 65].

*cr/20β-hsd* was identified as the most significant over expressed gene in females. This gene is known to be part of the oxidoreductase pathway for oocyte maturation preceding the enzymatic activity of *cyp19* (cytochrome P450 aromatase) [66]. *cyp19a1a* was proposed as the major gene for female determination in zebrafish [56]. Additional overexpressed genes in females such as *rtn4ip1*, *impa1, socs7* and *zp3* are involved in anti-apoptotic activity, embryonic development and fertility, prolactin and Jak-STAT signaling pathway and ovary development, respectively [67–70].

We found several cases of CNV within the set of genes that were differentially expressed between genders. qPCR analysis with gDNA has validated the existence of sexually dimorphic CNV in at least four genes, e.g., *cr/20βhsd*, *tspan8*, *gpa33* and *amh*. All four genes showed increased copy number in the direction of overexpression, in accordance with the reported positive correlation between relative expression level and gene dosage [71]. Five additional differentially expressed genes; *fcgrt*, *socs7*, *urgcp*, *zbed3* and *LOC100690239;* with multiple representations in the genome have been identified. CNV and dosage sensitivity have been hypothesized as evolutionary conserved factors of SD and SD plasticity among related species [72, 73]. Hattori et al. [60] suggested that master determinants of SD are predominantly recruited from the duplication of genes involved in the sex differentiation cascade. Our findings, supported by the above studies, indicate that CNV is a common feature of genes participating in SD and may be the alternative genomic structure to sex chromosome systems in fish.

The emerging significance of miRNAs in developmental processes and their ability to regulate large numbers of genes indicate their potential role in determining the onset of SD. We found 704, 668 and 636 miRNAs in tilapia embryos at 2, 5 and 9 dpf. Our findings of nine sexually differentially expressed miRNAs from 2 dpf illustrate their possible role in the early developing embryo. pma-mir-4585 was up-regulated by five and two fold in male *vs*. female embryos at 2 and 5 dpf, respectively, and to a lesser extent of 1.5 fold at 9 dpf. This decay of expression over time may indicate the significance of this miRNA in males soon after fertilization. For pma-mir-4585, an up-regulated miRNA in males, all six predicted target genes, including *cr/20β-hsd* that is known to activate female determination, were down-regulated in males (Table 3). The probability that this should occur by chance is (1/2)^6^ = 0.015, thus strengthening the functional targeting of these genes. Only this miRNA showed significantly perfect inverse expression correlation with its targeted genes, in accordance with the expected inhibition of mRNA translation of target genes [74]. Interestingly, this miRNA was firstly identified in sea lamprey (*Petromyzon marinus*) brain, which may be relevant to regulation of SD in fish through its potential activity in the brain.

Two of the nine miRNAs that were up-regulated in males, were conserved among vertebrates and thus allowed a thorough genomic analysis for their host genes; mir-218 was found to reside on *drg1* gene which had the highest expression in zebrafish testis, as compared to all other tissues tested [50], and mir-21 was localized to the *tubd1* gene which is involved in the elongation of the spermatid through a specialized microtubule system present during reshaping of the sperm head [75]. The function of this gene is related to the Sertoli pathway. Thus, the functions of both host genes in testis is consistent with the findings that miRNAs are usually coordinately coexpressed with their host genes mRNAs, implying that they derive from a common primary transcript [76]. Papagiannakopoulos et al. [77] reported that mir-21 targets several genes in the TGF-β and apoptosis pathways. Further investigation of the functions of the differentially expressed miRNAs in SD of tilapia is warranted.

We found that two thirds of the genes were highly expressed in females, especially at the early developing embryo, i.e. 2 to 5 dpf, and that two thirds of the miRNAs were up-regulated in males at the same period of embryonic development. Histological sex differentiation of the gonads in Patagonian pejerrey showed that the ovary differentiated at 3–4 weeks after hatching, as compared to 5–6 weeks in testis [60]. Based on these observations, it may be postulated that early onset of genes in the female cascade determine female, unless they are down-regulated by miRNAs, thus initiating the male determining pathway.

## Conclusions

**Figure 7:**
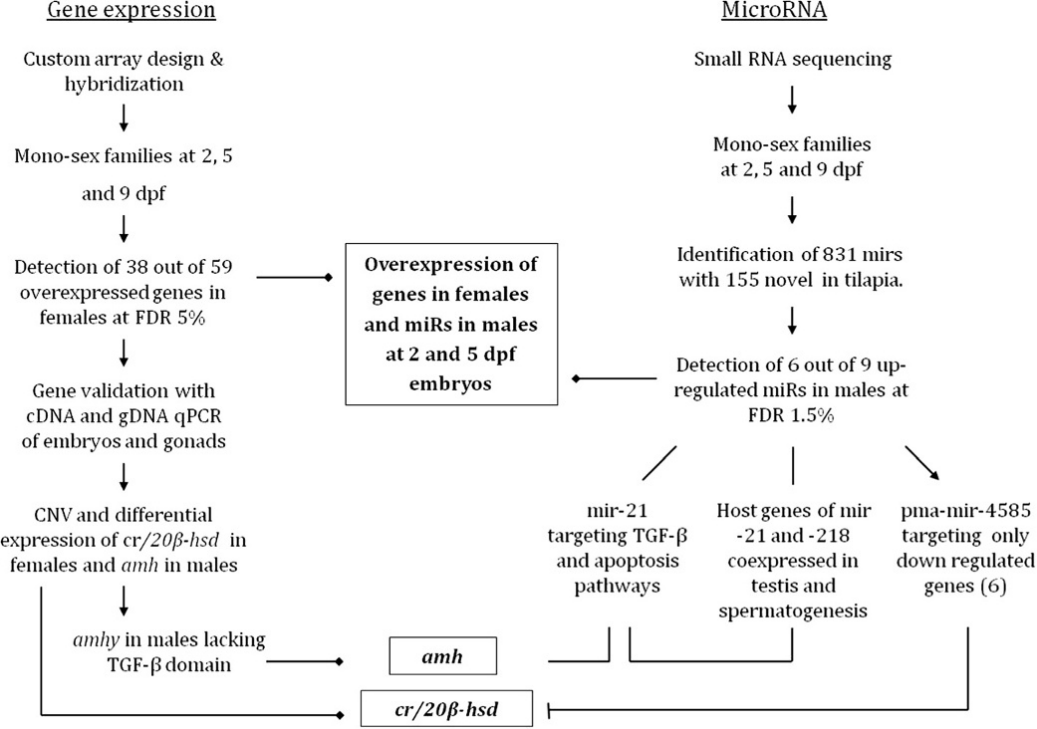
**Experimental workflow for detection of genes and miRNAs affecting SD and sex differentiation.** The differentially expressed genes and miRNAs between genders and their integrative role in SD and sex differentiation are displayed. The gene expression analysis is presented on the left axis, and the miRNAs analysis on the right. The implicated conclusions integrating the two streams of data are presented in the middle. SD: Sex determination, mir: miRNA precursor, miR: expressed 3p/5p mature sequence from miRNA precursors, *amh*: Anti-Müllerian hormone, *cr/20β-hsd*: carbonyl reductase 20 beta-hydroxysteroid dehydrogenase, TGF-β: transforming growth factor beta, FDR: false discovery rate, CNV: copy number variation*.* Experimental flow;  Implicated from the results;  Implicated from the literature;  Negative regulation.

In summary, this study reports the first discovery of sexually differentially expressed genes, genes enriched for CNV and apoptosis, miRNAs and their predicted gene targets and hosts that are functional from 2 dpf embryos. The experimental workflow used in this study is presented in Figure 7. The gene expression analysis is presented on the left axis along with the miRNAs analysis on the right. Systems biology techniques were used to derive and connect information on genes and miRNAs. The implicated conclusions integrating the two streams of data are presented in the middle of this Figure. *amh* and *cr/20β-hsd* genes may be involved in male and female determination and differentiation, respectively, similar to the proposed SD model for zebrafish [56]. *amhy* segregated in full concordance with the SD-linked marker on LG23 signifying the QTL for SD [21], thus indicating its potential role in SD.

To test the role of potential sex determining genes, miRNA and their predicted gene targets that were found in the current study for tilapia, targeted strategies should be considered, such as (i) mutant detection in candidate genes, as performed in zebrafish [56]; (ii) gene silencing using the TALEN or CRISPR/CAS9 technologies, as applied in tilapia and zebrafish, respectively [78, 79]; and (iii) transgenesis which was demonstrated for Nile tilapia [80, 81] *. amh* is a highly prioritized candidate gene for analysis by the variety of suggested methods in order to unravel its potential role in SD of tilapia.

## Electronic supplementary material

Additional file 1: Table S1: Annotations of 59 differentially expressed probes. (XLS 45 KB)

Additional file 2: Figure S1: PCA analysis for microarray expression data. (DOC 412 KB)

Additional file 3: Figure S2: False discovery rate for microarray expression data. (DOC 74 KB)

Additional file 4: Figure S3: Distribution of log transformed miRNAs expression data at 9 dpf. (DOC 58 KB)

## Abbreviations

SD: Sex determination
CNV: Copy number variation
dpf: Days post fertilization.
