# Supplementary material for: Identification of male-specific amh duplication, sexually differentially expressed genes and microRNAs at early embryonic development of Nile tilapia (Oreochromis niloticus)
Source: BMC Genomics. 2014 Sep 9;15(1):774. doi: 10.1186/1471-2164-15-774 (PMC4176596; doi:10.1186/1471-2164-15-774)
Supplement: Supplementary file 2 — Additional file 2: Figure S1: PCA analysis for microarray expression data. (DOC 412 KB) [file 12864_2014_6466_MOESM2_ESM.doc]

**Additional file 2: Figure S1:** Individual biological samples relative to the first 3 factors of the principle component analysis for microarray expression data of 43,803 probes hybridized with cDNA of 56 biological samples of predetermined gender at 2, 5 and 9 days post fertilization. Locations of males and females are denoted by ♂ and ♀ symbols, in red, blue and green for two, five and nine days post fertilization, respectively.

**
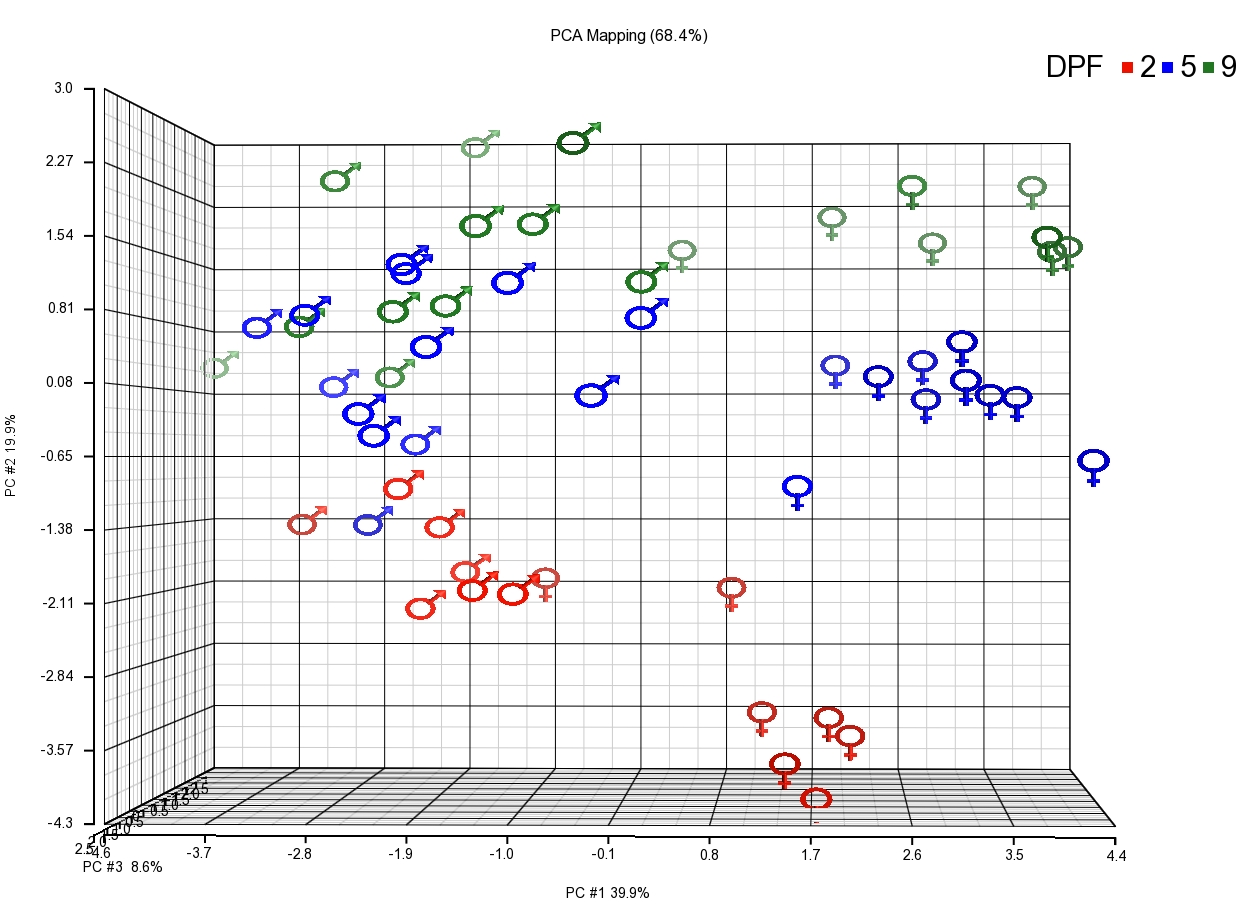
**
