# Supplementary material for: Identification of male-specific amh duplication, sexually differentially expressed genes and microRNAs at early embryonic development of Nile tilapia (Oreochromis niloticus)
Source: BMC Genomics. 2014 Sep 9;15(1):774. doi: 10.1186/1471-2164-15-774 (PMC4176596; doi:10.1186/1471-2164-15-774)
Supplement: Supplementary file 3 — Additional file 3: Figure S2: False discovery rate for microarray expression data. (DOC 74 KB) [file 12864_2014_6466_MOESM3_ESM.doc]

**Additional file 3: Figure S2:** False discovery rate (FDR, %) as a function of nominal significance values (P value) and number of significant probes.

**
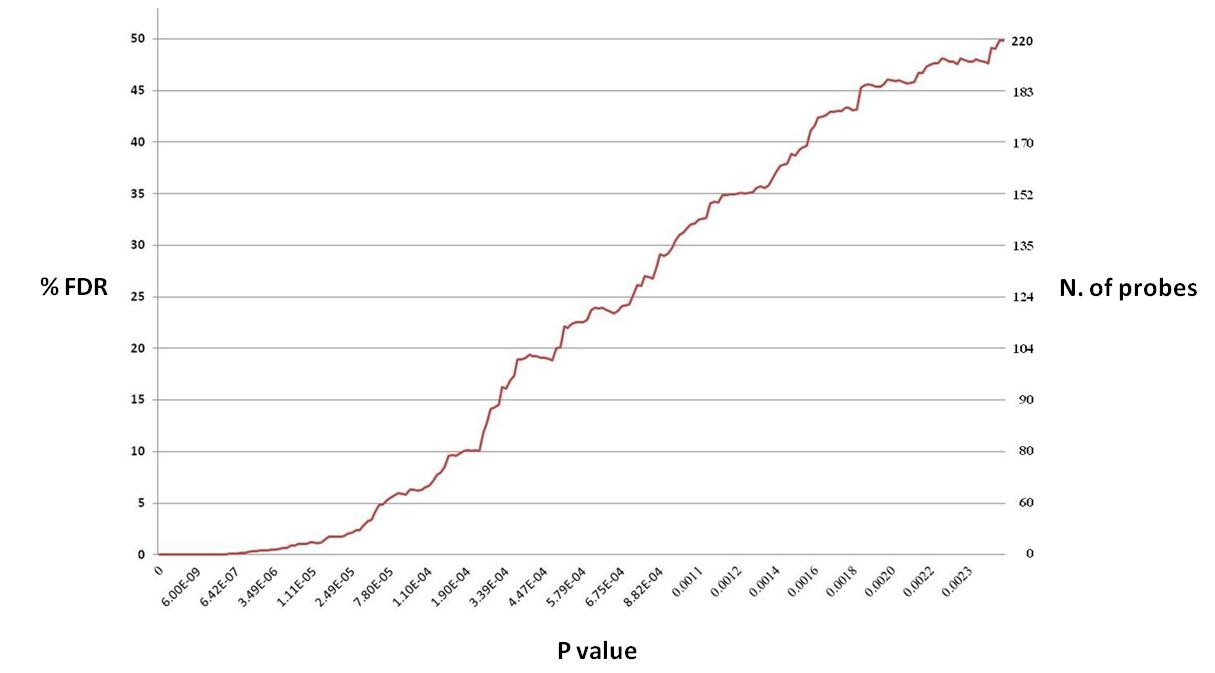
**
