# Supplementary material for: Identification of male-specific amh duplication, sexually differentially expressed genes and microRNAs at early embryonic development of Nile tilapia (Oreochromis niloticus)
Source: BMC Genomics. 2014 Sep 9;15(1):774. doi: 10.1186/1471-2164-15-774 (PMC4176596; doi:10.1186/1471-2164-15-774)

**Additional file 4: Figure S3:** Distribution of log transformed miRNAs expression between genders of 972 mature and star sequences of ≥ 4 reads at 9 dpf. Red squares indicate miRNAs that are differentially expressed between genders by > 4 standard deviations.


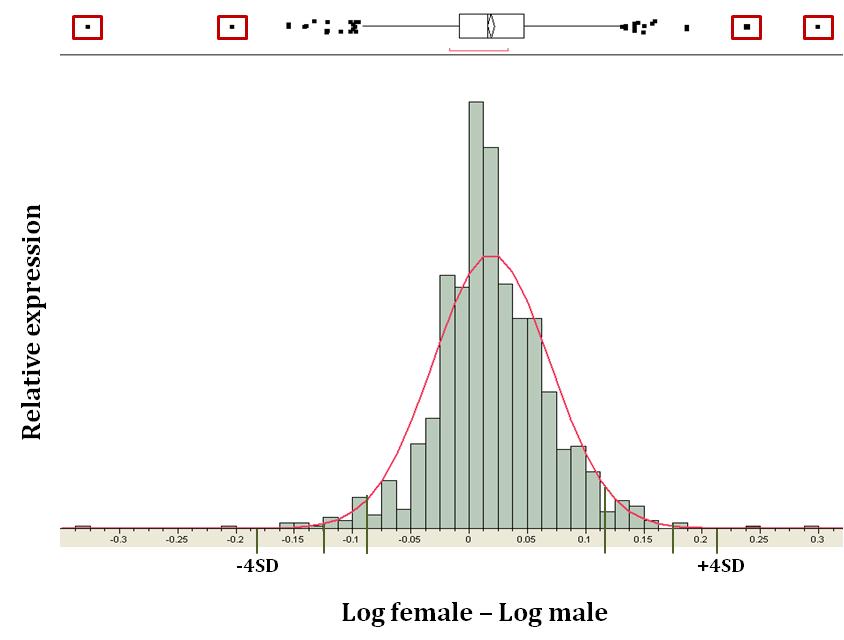

Supplement: Supplementary file 4 — Additional file 4: Figure S3: Distribution of log transformed miRNAs expression data at 9 dpf. (DOC 58 KB) [file 12864_2014_6466_MOESM4_ESM.doc]
